# Structural basis of the therapeutic anti-PD-L1 antibody atezolizumab

**DOI:** 10.18632/oncotarget.21652

**Published:** 2017-10-06

**Authors:** Fei Zhang, Xiaoqiang Qi, Xiaoxiao Wang, Diyang Wei, Jiawei Wu, Lingling Feng, Haiyan Cai, Yugang Wang, Naiyan Zeng, Ting Xu, Aiwu Zhou, Ying Zheng

**Affiliations:** ^1^ Hongqiao International Institute of Medicine, Shanghai Tongren Hospital/Faculty of Basic Medicine, Key Laboratory of Cell Differentiation and Apoptosis of The Chinese Ministry of Education, Shanghai Jiao Tong University School of Medicine, Shanghai, China; ^2^ Faculty of Basic Medicine, Nanjing Medical University, Nanjing, China; ^3^ The Therapeutic Antibody Research Center of SEU-Alphamab, Southeast University, Nanjing, China

**Keywords:** atezolizumab, PD-L1, crystal structure, immunotherapy, hot-spot residue

## Abstract

Monoclonal antibodies targeting PD-1/PD-L1 signaling pathway have achieved unprecedented success in cancer treatment over the last few years. Atezolizumab is the first PD-L1 monoclonal antibody approved by US FDA for cancer therapy; however the molecular basis of atezolizumab in blocking PD-1/PD-L1 interaction is not fully understood. Here we have solved the crystal structure of PD-L1/atezolizumab complex at 2.9 angstrom resolution. The structure shows that atezolizumab binds the front beta-sheet of PD-L1 through three CDR loops from the heavy chain and one CDR loop from the light chain. The binding involves extensive hydrogen-bonding and hydrophobic interactions. Notably there are multiple aromatic residues from the CDR loops forming Pi-Pi stacking or cation-Pi interactions within the center of the binding interface and the buried surface area is more than 2000 Å^2^, which is the largest amongst all the known PD-L1/antibody structures. Mutagenesis study revealed that two hot-spot residues (E58, R113) of PD-L1 contribute significantly to the binding of atezolizumab. The structure also shows that atezolizumab binds PD-L1 with a distinct heavy and light chain orientation and it blocks PD-1/PD-L1 interaction through competing with PD-1 for the same PD-L1 surface area. Taken together, the complex structure of PD-L1/atezolizumab solved here revealed the molecular mechanism of atezolizumab in immunotherapy and provides basis for future monoclonal antibody optimization and rational design of small chemical compounds targeting PD-L1 surface.

## INTRODUCTION

Cancer immunotherapies which utilize antibodies masking the inhibitory receptor have drawn considerable attention in recent years [1–4]. Several monoclonal antibodies (MAbs) targeting CTLA-4, PD-1 and PD-L1 have been approved by FDA for clinical applications in USA. PD-L1 (CD274, B7-H1) is expressed widely on both lymphoid and nonlymphoid tissues [5] and it is the primary PD-1 ligand. It is up-regulated in solid tumors, where it can inhibit the activity of PD-1^+^, tumor-infiltrating CD4^+^ and CD8^+^ T cells [6–8]. Blockade of PD-L1 binding is an attractive strategy for restoring tumor-specific T-cell immunity [9, 10]. Tumor responses have been obtained both with anti-PD-1 and anti-PD-L1 therapies in patients with several forms of cancer [11–15].

Atezolizumab (TECENTRIQ) developed by Genetech is the first therapeutic anti-PD-L1 antibody approved in the United States for the treatment of patients with metastatic urothelial carcinoma or non-small cell lung cancer who have progressed during or following platinum-containing chemotherapy [https://www.fda.gov/NewsEvents/Newsroom/Press Announcements/ucm501762.htm]. There are more than forty ongoing trials with atezolizumab either as monotherapy or in combination with other agents (e.g., bevacizumab, cobimetinib, obinutuzumab, bendamustine, ipilimumab, interferonalfa). Clinical indications under investigation include renal cell carcinoma, hepatocellular carcinoma, triple negative breast cancer, colorectal cancer, hematologic malignancies in addition to other tumor types as well. Several other anti-PD-L1 antibodies including avelumab, durvalumab and KN035 are also in intensive clinical trials. Crystal structures of these PD-L1 antibodies such as avelumab, durvalumab, BMS936559 and KN035 have been reported recently [16–19], however, it is unclear how atezolizumab binds and blocks PD-1/PD-L1 pathway. In the present study, we have prepared the Fab fragment of atezolizumab and solved its crystal structure in complex with PD-L1 and analyzed its binding characteristics.

## RESULTS AND DISCUSSION

### Overall structure of PD-L1/atezolizumab complex

**Figure 1:**
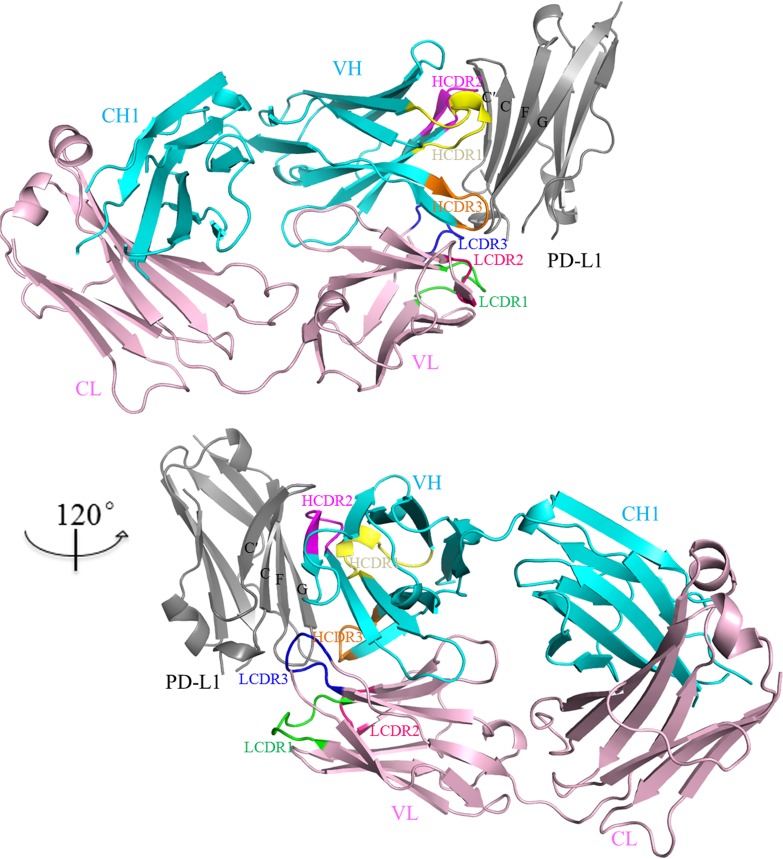
Overall structure of PD-L1/atezolizumab complex The IgV domain of PD-L1 is shown in grey and the heavy chain and light chain of atezolizumab are shown in cyan and pink respectively. The CDR loops from the heavy chain are colored in yellow (HCDR1), magenta (HCDR2) and orange (HCDR3) respectively. The CDR loops from the the light chain are colored in green (LCDR1), hot pink (LCDR2) and blue (LCDR3) respectively. Atezolizumab binds the front β-sheet of PD-L1-IgV domain (grey) through three CDR loops from the heavy chain and CDR3 loop (LCDR3) from the light chain.

**Figure 2:**
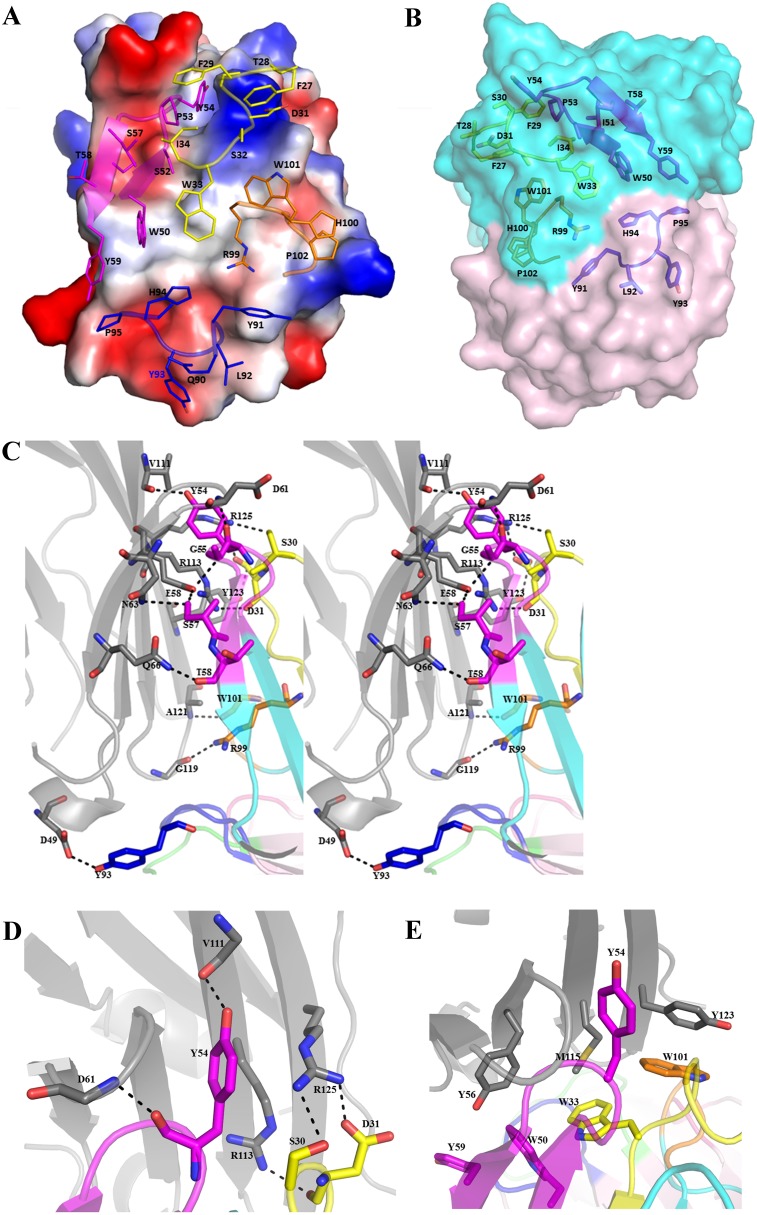
Detailed interactions in the interface of PD-L1/atezolimumab complex The electrostatics surface of PD-L1 is shown in **(A)** with key residues from CDR loops of atezolizumab shown in sticks. The CDR loops are color with same scheme as in Figure 1 where HCDR1 is in yellow, HCDR2 in magenta, HCDR3 in orange and LCDR3 in blue. The binding surface of atezolizumab is shown in **(B)** with heavy chain in cyan and light chain in magenta. Some of the key residues of heavy and light chain are in sticks **(B)**. The wall-eye stereo view of the interface of PD-L1/atezolizumab complex shows detailed hydrogen bonds between these two molecules **(C)**. Residues involved are shown as sticks with hydrogen bonds as dashed lines.Y54 of HCDR2 is inserted into a cleft formed by CC’ loop and strand G of PD-L1 forming a hydrogen bond with V111 of PD-L1 which is further stabilized by the side chain of R125 of PD-L1 through cation-Pi interaction. The side chain of R125 is in turn stabilized by S30 and D31 from HCDR1 loop **(A, D)**. PD-L1 residues of M115 along with Y56 and Y123 form hydrophobic interactions with a hydrophobic surface formed aromatic residues W33, W50, Y59 and W101 of CDR loops of atezolizumab **(E)**.

Atezolizumab Fab fragment was expressed in HEK293 expression system and purified from the culture medium. The IgV domain of PD-L1 was expressed in *E.coli* as inclusion body and purified after refolding as previously described [19]. Crystals of the PD-L1/atezolizumab complex were grown from 2 M ammonium sulfate in 0.1 M Tris pH7.0 and the structure of this complex was solved at 2.9Å resolution with a single complex assembly in the asymmetric unit (Table 1). PD-L1 assumes a beta-sandwich immunoglobulin-variable (IgV)-type topology with Cys40 and Cys114 forming a disulfide bridge. The binding site of atezolizumab on PD-L1 is mainly located on the front β-sheet which is constituted by strands A, G, F, C, and C’ of the IgV domain of PD-L1 (Figure 1). The structure revealed that both heavy chain (VH) and light chain (VL) of atezolizumab interact with PD-L1. All three complementarity determining region (CDR) loops of VH of atezolizumab are involved while only CDR3 loop of VL forms interaction with PD-L1 (Figure 2). When structure of PD-L1/atezolizumab complex is superimposed with the structure of full length PD-L1 (PDB: 5JDR) or PD-1/PD-L1 complex (PDB: 4ZQK), the root-mean-square deviations (RMSDs) are 0.53 Å and 0.56 Å respectively, indicating no significant changes in PD-L1 structure during atezolizumab binding (Supplementary Figure 1) [19, 20].

**Table 1:** Crystallographic data collection and refinement statistics

	PD-L1/atezolizumab complex
***Data collection:***	
**Beamline**	SSRF 17U
**Space group**	C121
**Cell dimensions**	
**a, b, c (Å)α, β, γ (°)**	127.43, 73.05, 64.0090.00, 104.39, 90.00
**Wavelength (Å)**	0.9792
**Resolution (Å)**	2.90-61.60 (2.90-3.08)
**Total NO. of observation**	34659(5741)
**Total NO. unique**	11768 (1927)
**R_merge_ (%)**	20.0 (57.8)
**I/σI**	4.6 (1.8)
**Completeness (%)**	93.4 (95.1)
**Multiplicity**	2.9 (3.0)
***Refinement:***	
**Resolution (Å)**	2.90-61.60
**No. of reflections/free**	11744/545
**No. of residues**	518
**No. of atoms**	3979
**R_work_/R_free_**	0.239/0.295
**B-factors (Å^2^)**	45.7
**RMSD**	
** Bond lengths (Å) Bond angles (°)**	0.0040.845
**Ramachandran plot**	
** In Preferred Region (%)**	94.6
** In Allowed Region (%)**	5.16
** Outliers (%)**	0.2

### Interactions between PD-L1 and atezolizumab

**Figure 3:**
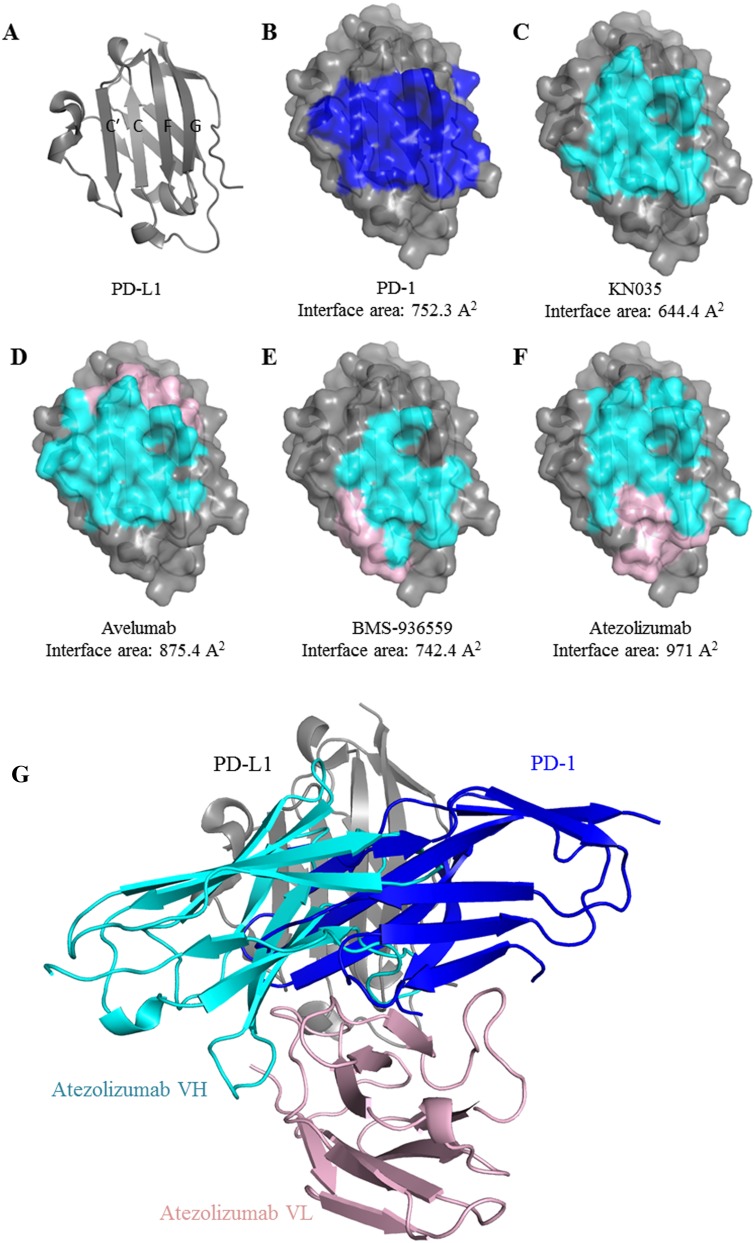
Comparison of the binding mode of anti-PD-L1 MAbs The front β-sheet of PD-L1 is composed with strand C’, C, F and G **(A)**. The binding surface area that is covered by PD-1 **(B)**, KN035 **(C)**, Avelumab **(D)**, BMS-936559 **(E)** or Atezolizumab **(F)** is highlighted with the covered surface area calculated. The areas covered by heavy or light chain of MAbs **(D, E, F)** are color in cyan and pink respectively. Superposition of the structure of PD-L1/atezolizumab complex with PD-1/PD-L1complex (PDB: 4ZQK) shows clashes between PD-1 and atezolizumab indicating that the antibody competes with PD-1 for binding to PD-L1. the CH1domain of heavy chain and CL domain of light chain were omitted **(G)** for clarity.

Detailed structural analysis of PD-L1/atezolizumab complex by PISA software (Proteins, Interfaces, Structures and Assemblies) shows that there are 13 hydrogen bonds (Supplementary Table 1) and about 82 contacts within 3.7Å radius in PD-L1/atezolizumab interface (Supplementary Table 2). PD-L1 residues from the strand C (D49, A52, I54, Y56, E58) and strand C’(N63, Q66, V68), the C'C loop (M59, E60, D61, K62), strand F (V111, R113, M115), FG loop (G119) as well as strand G (A121, D122, Y123, R125) form an extended surface to interact with heavy chain residues from CDR1 (S30, D31), CDR2 (Y54, G55, S57, T58), CDR3 (R99, W101) loops from the heavy chain of atezolizumab (Figure 2A, 2B). Especially, residues (Y54, G55, S57, T58) of the CDR2 loop of the heavy chain (HCDR2) form 6 hydrogen bonds with residues from strand C (E58), strand C’ (N63, Q66) and CC’ loop (D61) (Figure 2C), and residues from HCDR1 loop (S30, D31) form 4 polar interactions with strand F (R113) and strand G (Y123, R125) (Figure 2D). Notably the side chain of Y54 from HCDR2 loop pokes into a cleft formed by strand C and G and forms a H-bond with the main-chain oxygen atom of V111 of PD-L1, and at the same time it is further stabilized by a cation-Pi interaction with the side chain of R125 (Figure 2D). Furthermore the side chain of M115 of strand F in PD-L1 is positioned along the hydrophobic patch formed by W33 (HCDR1), W50 (HCDR2) and W101 (HCDR3) of atezolizumab, while the adjacent Y123 of strand G packs with W101 (HCDR3) through π-π interaction (Figure 2E). Residues L92 and Y93 from LCDR3 form close contact with PD-L1 residues A51, A52 and G119. Overall, these extensive H-bonding and hydrophobic interactions result a total buried surface area of more than 2000 Å^2^ in PD-L1/atezolizumab binding interface, which is the largest among the known PD-L1/antibodies structures. The buried surface area of atezolizumab is 1029 Å^2^ with 784 Å^2^ from the heavy chain and 245 Å^2^ from the light chain in the binding interface, and the buried surface area of PD-L1 is 971 Å^2^ (Figure 3A). Although the buried surface area is not necessarily proportional to the binding affinity between the antibody and antigen, the large binding surface of atezolizumab may provide certain advantage in the selectivity of this antibody.

### Structural basis of atezolizumab in PD-1/PD-L1 blockade

To analyze how atezolizumab would block PD-L1's interactions, we systemically analyzed the binding areas of PD-L1 when it is complexed with its ligand or blocking antibodies. Interestingly we found that all the binding areas on PD-L1 on located on the front beta-sheet with a buried surface area of 752.3 Å^2^ for PD-1, 644.4 Å^2^ for KN035, 875.4 Å^2^ for avelumab, 742.4 Å^2^ for BMS-936559 and 971 Å^2^ for atezolizumab respectively. These binding areas largely overlap each other (Figure 3B). Also it appears that the heavy chain rather than the light chain in all the MAbs plays a dominant role in binding (Supplementary Figure 2). Furthermore when the structure of PD-1/PD-L1 complex (PDB: 4ZQK) is overlaid with that of PD-L1/atezolizumab complex, it is apparent that atezolizumab clashes with PD-1 and they could not bind to a same PD-L1 molecule at the same time. Therefore, atezolizumab, like other known PD-L1 antibodies, blocks PD-1/PD-L1 interaction through competing with PD-1 for the same surface area on PD-L1.

Despite these common features, there are distinct differences amongst these PD-L1 antibodies. For example, the relative positions of the heavy and light chain of the MAbs when bound on PD-L1 differ significantly. The heavy and light chains of avelumab adopt a bottom-top configuration, where the heavy chain binds the bottom half of the PD-L1 surface and the light chain binds on the top part. BMS-936559 antibody adopts a right-left configuration with atezolizumab adopting a top-bottom configuration. Notably the anti-PD-L1 nanobody KN035 binds PD-L1 mainly through a single CDR loop of a single IgV domain. Also some of these antibodies are in either IgG1 form (avelumab, durvalumab, atezolizuamb) or IgG4 isoform (BMS-936559) [21]. These differences will result differences in the orientations of these antibodies when bound on PD-L1 on the cell membrane and affect subsequent binding of Fc receptors, which will lead to differences in antitumor activities or selectivity towards different types of cancers. Obviously extensive clinical tests are required to make full use of these antibodies for the benefits of cancer patients.

It is also somewhat surprising that all these PD-L1 blocking antibodies bind PD-L1 on the flat front beta-sheet as generally targeting a relatively flat protein surface is very challenging. When compared with other immune checkpoint blocking antibodies, we have found that CTLA-4 blocking antibodies such as ipilimumab (PDB:5TRU) and tremelimumab (PDB:5GGV) similarly target the front surface of IgV domain of CTLA-4, while PD-1 blocking antibodies such as pembrolizumab (PDB:5GGS) and nivolumab (PDB:5GGR) mainly bind PD-1 on the connecting loops of the front beta-sheet [18, 22–24]. It is unclear if the differences in the epitopes of these receptors are due to the differences in antibody screening processes or due to differences in the geometry and electrostatic properties of the IgV domains of these receptors. Nevertheless, the distinct binding surfaces of these PD-L1 antibodies targeting same area on PD-L1 revealed by these crystallography studies provide invaluable test cases for fine-tuning programs in predicting protein-protein interactions.

### Hot-spot residues

**Figure 4:**
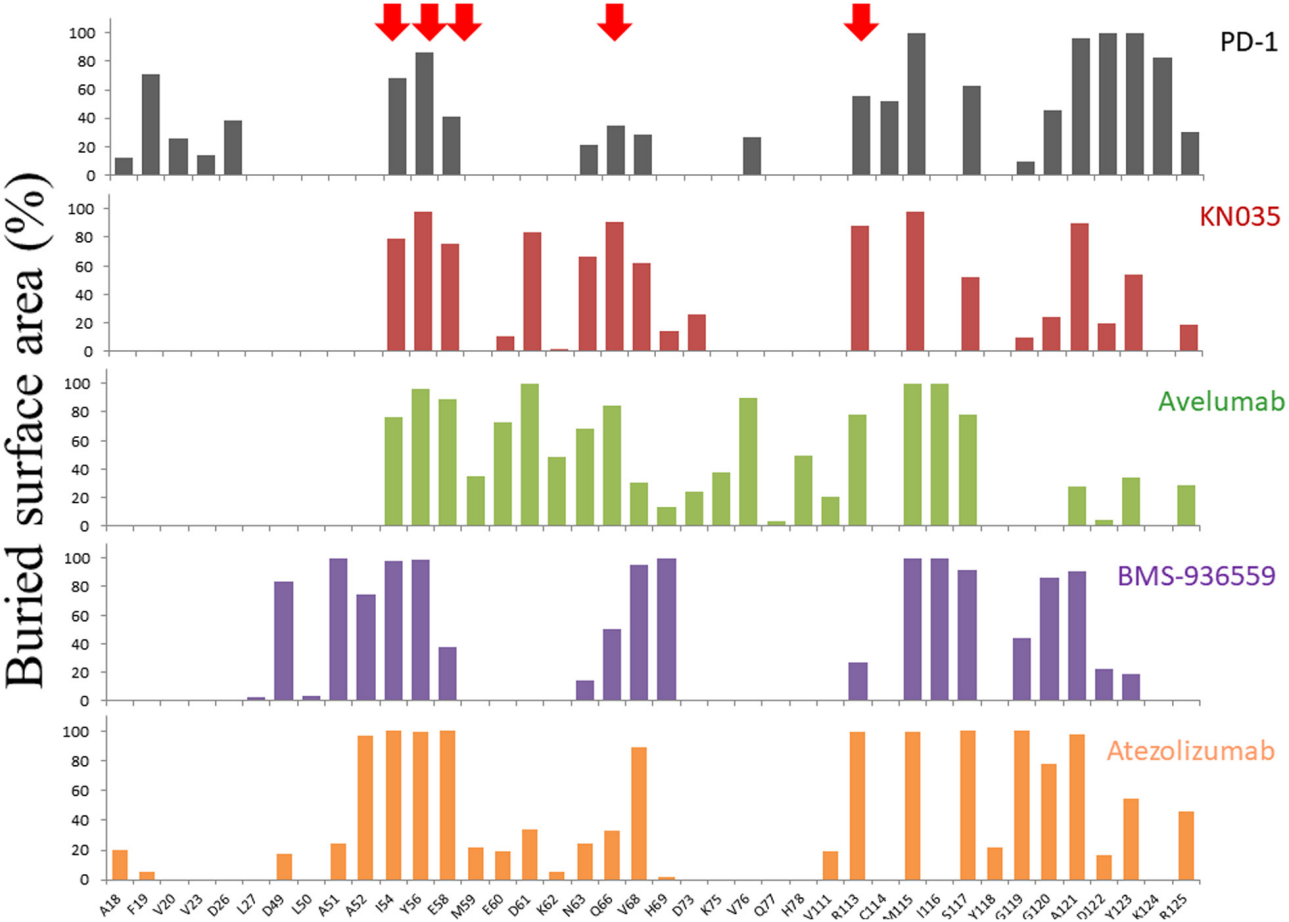
Buried surface areas of residues in PD-L1 binding interface All the PD-L1 residues that are involved in binding to PD-1, KN035, Avelumab, BMS-936559 and Atezolizumab are selected with the percentage of the buried surface area of each residue plotted. The five hot-spot residues (I54, Y56, E58, Q66 and R113) identified from PD-L1/KN035 structure are labeled with red arrows. Although the structure of PD-L1/Durvalumab was also published recently, the coordinate of this structure has not been released and is not included here for comparison.

It has been shown that many residues are often involved in a protein binding interface, but only a few of them make critical contributions towards complex formation. These key residues are often called the hot-spot residues of a given protein surface and they are general targets for rational drug design in blocking protein interactions [25, 26]. We have previously solved the crystal structure of an anti-PD-L1 nanobody KN035 and analyzed the contribution of each PD-L1 residue of the interface towards binding through mutagenesis and affinity measurement. The hot-spot residues of PD-L1 surface identified are I54, Y56, E58, Q66 and R113. Here we have compared the binding interfaces of all known PD-L1/antibody complexes and calculated the buried surface area of each residue in the interfaces (Figure 4). The plot shows that the binding of PD-L1 towards each antibody involves about 20-30 residues with about half of these residues significantly covered by the antibodies in the interfaces. Most significantly, the five hot-spot residues we identified are all involved in binding to these antibodies. Subsequently we tested the role of these hot-spot residues in atezolizmab binding. The PD-L1 mutants, prepared in our previous study [19], were tested for their binding affinity towards atezolizumab (Table 2). The results showed that replacement of hot-spot residues (E58 and R113) by alanine leads to 18 and 9-fold decrease in the binding affinity of PD-L1 towards atezolizumab respectively, while other residues play a relatively minor role. This indicates that atezolizumab adopts two of the hot-spot residues from this part of PD-L1 surface for its efficient binding. Further confirmation of the importance of these PD-L1 residues comes from the recent structural study of PD-L1 with chemical PD-L1 inhibitors developed by Bristol-Myers Squibb where binding of these compounds on PD-L1 involves two hot-spot residues I54 and Y56 [27, 28]. Although it is largely unexpected that these chemical inhibitors inhibit PD-L1 binding surface through inducing dimerization of PD-L1, the hot-spot residues shown here will provide rational targets for the development of new class of chemical compounds as PD-L1 inhibitors.

**Table 2:** Binding affinities of PD-L1 variants towards KN035 and atezolizumab

PD-L1 variants	K_d_ of KN035 (M)	K_d, varitant_/K_d, WT_	K_d_ of atezolizumab (M)	K_d, variant_/K_d, WT_
WT	3.0E-09	1	9.96E-09	1
I54A	2.42E-07	80.7	3.23E-08	3.2
Y56A	1.24E-06	413.3	2.68E-08	2.7
E58A	1.49E-07	49.7	1.81E-07	18.2
D61A	1.99E-08	6.6	9.99E-09	1.0
N63A	2.30E-08	7.7	1.73E-08	1.7
Q66A	4.88E-07	162.7	2.46E-09	0.25
R113A	5.34E-07	178	8.52E-08	8.6
M115A	5.51E-08	18.4	4.57E-08	4.6
Y123A	4.24E-08	14.1	4.66E-08	4.7
R125A	2.97E-08	9.9	5.89E-08	6.0

The binding affinity was determined where KN035-Fc or full-length atezolizumab was immobilized on a protein A chip and then treated with different concentrations of PD-L1 variants as previously described [19].

In conclusion, the crystal structure of therapeutic antibody atezolizumab complexed with PD-L1 solved here shows that the antibody blocks PD-1/PD-L1 pathway through competing with PD-1 for the binding surface on PD-L1 and this provides basis for further antibody optimization and for the development of novel chemical PD-L1 inhibitors for immunotherapy.

## MATERIALS AND METHODS

### Protein purification and complex preparation

The Fab fragment of atezolizumab was expressed in the transient HEK293 expression system. Mixture of PEI (Polyscience) and expression plasmids coding the heavy chain and light chain of the Fab fragment of atezolizumab (w/w 3:1) was transfect into HEK293 suspension cell at a density of 4^*^10^6^/ml. Culture medium were collected after 3 days through centrifugation. The assembled Fab fragment of atezolizumab with a his-tag at the C-terminus of the heavy chain was purified by Ni column (GE healthcare) from the medium after dialysis against 10 mM Tris-HCl, pH 7.4, 150 mM NaCl. IgV domain of PD-L1 is prepared as previously described [19]. Briefly, the DNA sequence encoding human PD-L1 IgV domain (amino acids 19-132) with a C-terminal His-tag was cloned into pET-28a and transformed into *E.coli* BL21 (DE3) as inclusion bodies. Cells were cultured at 37°C in LB and induced with 0.5 mM IPTG (isopropyl-β-D-thiogalactoside) once the optical density at 600 nm reached 1.0. After a further 16-hour incubation at 37°C, the cells were collected by centrifugation, resuspended in lysis buffer (20 mM Tris-HCl, pH 7.4, 1% Triton X-100, and 20 mM EDTA) and lyzed by sonication. Inclusion bodies were recovered by centrifugation at 15,000 g for 10 minutes and were then washed 3 times with lysis buffer, followed by washing with buffer without Triton X-100. The inclusion bodies were dissolved in 6 M GuHCl, 0.5 mM EDTA, and 10 mM DTT, 20 mMTris, pH 7.4 and added drop-wise into a refolding buffer consisting of 1 M Arg hydrochloride, 0.1 M Tris, pH 8.0, 2mM EDTA, 0.25 mM oxidized glutathione, 0.25 mM reduced glutathione and 0.1 mg/ml of atezolizumab. The PD-L1 IgV domain/atezolizumab complexes were subsequently purified using ion exchange and gel filtration columns (GE Healthcare).

### Crystallization of PD-L1/atezolizumab complex

PD-L1/atezolizumab complex was concentrated to ~10 mg/ml and screened for crystallization conditions using commercially available buffers with sitting-drop vapor diffusion method where 0.2 μl of the protein solution was mixed with 0.2 μl of reservoir solution. Diffraction-quality crystals of PD-L1/atezolizumab complex were obtained at room temperature from 2 M ammonium sulfate and 0.1 M Tris pH7.0 after optimization.

### Structure determination and refinement

Crystals were cryoprotected in 20% glycerol in the mother liquor and flash-cooled in liquid nitrogen. Diffraction data were collected on beamlines BL17U at SSRF, Shanghai, China. The data were indexed and processed with iMosflm and scaled with Aimless from the CCP4 suite [29]. The structure was solved by Phaser [30] using PD-L1 and atezolizumab models derived from PDB entries 5JDR and 5GGT, respectively. The models were subsequently manually built using Coot and refined using PHENIX [31, 32]. Figures were produced with PyMOL software [33]. The atomic coordinates and the structure factors have been deposited in the Protein Data Bank with code 5XXY. The interface of protein structure was analyzed by PISA [34].

### Dissociation rate constant

The binding kinetics of PD-L1 variants to atezolizumab was measured as previously described [19]. Briefly, all sensors were activated in PBS with 0.1% w/v bovine serum albumin (BSA) by agitating 96-well microtiter plates at 1,000 rpm to minimize nonspecific interactions. Probes were soaked with 10 μg/ml atezolizumab for 40 seconds before equilibration for 60 seconds in PBS with 0.1% BSA. Variants of PD-L1 were prepared as a 2-fold serial dilution in PBS with 0.1% BSA and separately incubated with the atezolizumab bound on the tips for 120 seconds. Then, the PD-L1 variants were allowed to dissociate for up to 320 seconds. All measurements were corrected for baseline drift by subtracting a control sensor exposed to running buffer only. Data analysis and curve fitting were carried out using Octet software.

## SUPPLEMENTARY MATERIALS FIGURES AND TABLES




