# Supplementary material for: Structural basis of the therapeutic anti-PD-L1 antibody atezolizumab
Source: Oncotarget. 2017 Oct 6;8(52):90215–24. doi: 10.18632/oncotarget.21652 (PMC5685743; doi:10.18632/oncotarget.21652)
Supplement: Supplementary file 1 [file oncotarget-08-90215-s001.pdf]

Structural basis of the therapeutic anti-PD-L1 antibody atezolizumab

SUPPLEMENTARY MATERIALS

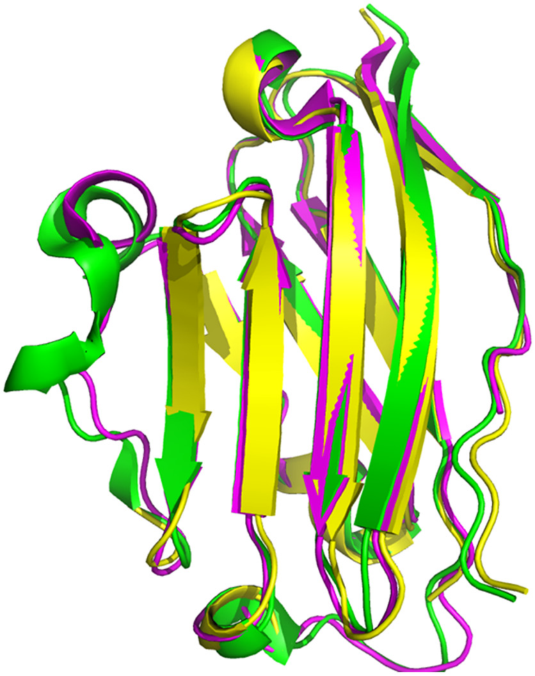

Supplementary Figure 1: Overlaid structures of the IgV domains of PD-L. The structures of PD-L1 IgV domains from PD-1/PD-L1 complex (PDB: 4ZQK, green), free PD-L1 (PDB: 5JDR, magenta), PD-L1/atezolizumab (yellow) are superposed indicating minor conformational changes during antibody binding.

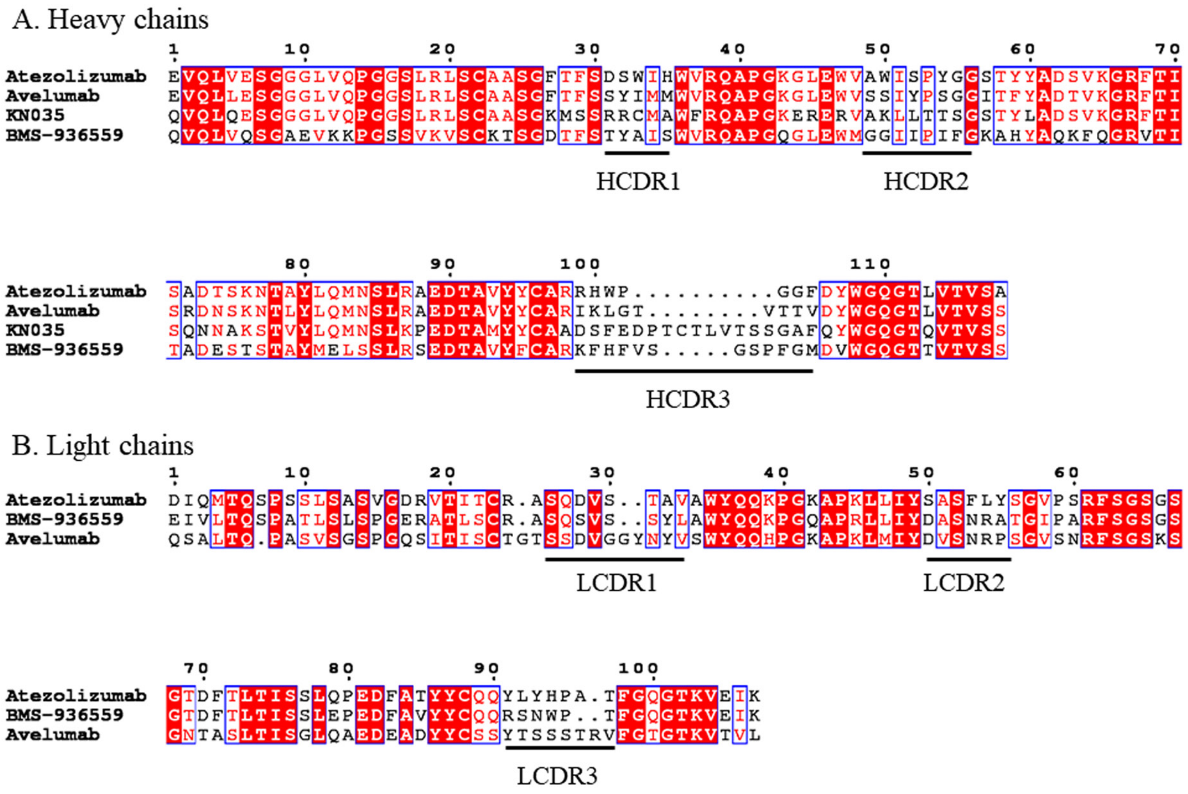

Supplementary Figure 2: Sequence alignment among atezolizumab, avelumab, KN035 and BMS-936559.

Supplementary Table 1: Polar interactions between PD-L1 and atezolizumab

PD-L1 contact residue	PD-L1 residue location	AtezolizumabVH contact residue	Atezolizumab VH residue location
E58	C strand	G55	HCDR2
E58	C strand	S57	HCDR2
D61	CC’ loop	T54	HCDR2
N63	C’strand	S57	HCDR2
Q66	C’ strand	T58	HCDR2
V111	F strand	Y54	HCDR2
R113	F strand	D31	HCDR1
G119	FG loop	R99	HCDR3
A121	G strand	W101	HCDR3
Y123	G strand	D31	HCDR1
R125	G strand	S30	HCDR1

PD-L1 contact residue	PD-L1 residue location	AtezolizumabVL contact residue	Atezolizumab VL residue location
D49	C strand	Y93	LCDR3

Supplementary Table 2: Contacts less than 3.7 Å observed between PD-L1 and atezolizumab

See Supplementary File 1
