# Supplementary material for: Structural basis of the therapeutic anti-PD-L1 antibody atezolizumab
Source: Oncotarget. 2017 Oct 6;8(52):90215–24. doi: 10.18632/oncotarget.21652 (PMC5685743; doi:10.18632/oncotarget.21652)
Supplement: Supplementary file 2 [file oncotarget-08-90215-s002.docx]

**Supplementary Table 2: Contacts less than 3.7 Å observed between PD-L1
and atezolizumab**

| Chain | Residue | Atom | Chain | Residue | Atom | Distance |
| --- | --- | --- | --- | --- | --- | --- |
| A | Y56 | CE1 | H | W50 | CZ3 | 3.41 |
| A |  |  | H | W50 | CH2 | 3.32 |
| A |  | CE2 | H | S57 | CB | 3.68 |
| A |  | OH | H | W50 | CZ3 | 3.32 |
| A |  |  | H | T58 | O | 3.64 |
| A |  |  | H | W50 | CH2 | 3.21 |
| A | E58 | CD | H | S57 | OG | 3.40 |
| A |  | OE1 | H | S52 | CB | 3.05 |
| A |  |  | H | G55 | N | 3.27 |
| A |  |  | H | G55 | CA | 3.15 |
| A |  |  | H | S57 | OG | 2.46 |
| A |  | OE2 | H | S52 | CB | 3.68 |
| A | M59 | O | H | Y54 | O | 3.41 |
| A |  |  | H | G55 | CA | 3.62 |
| A | E60 | CA | H | Y54 | O | 3.65 |
| A |  | C | H | Y54 | O | 3.63 |
| A | D61 | N | H | Y54 | O | 2.72 |
| A |  | CA | H | G55 | O | 3.51 |
| A |  |  | H | Y54 | O | 3.54 |
| A |  | C | H | G55 | O | 3.18 |
| A |  | O | H | G55 | O | 3.67 |
| A | K62 | N | H | G55 | O | 3.15 |
| A | N63 | ND2 | H | S57 | CB | 3.56 |
| A |  |  | H | S57 | OG | 3.54 |
| A | Q66 | CD | H | T58 | O | 3.62 |
| A |  | NE2 | H | T58 | N | 3.47 |
| A |  |  | H | T58 | C | 3.39 |
| A |  |  | H | T58 | O | 2.34 |
| A | V68 | CG1 | H | Y59 | CE1 | 3.63 |
| A |  |  | H | Y59 | CZ | 3.66 |
| A | V111 | C | H | Y54 | OH | 3.51 |
| A |  | O | H | Y54 | OH | 2.81 |
| A |  | CB | H | Y54 | OH | 3.21 |
| A |  | CG1 | H | Y54 | OH | 3.38 |
| A | R113 | CB | H | Y54 | CD2 | 3.59 |
| A |  |  | H | Y54 | CE2 | 3.55 |
| A | M115 | NH2 | H | D31 | O | 3.08 |
| A | G119 | CG | H | W101 | CZ3 | 3.56 |
| A | A121 | O | H | R99 | NH1 | 2.73 |
| A |  | N | H | R99 | NH1 | 3.50 |
| A |  |  | H | W101 | O | 2.94 |
| A |  | C | H | W101 | CE3 | 3.26 |
| A |  | O | H | W101 | CE3 | 3.16 |
| A |  |  | H | W101 | CA | 3.68 |
| A |  |  | H | W101 | C | 3.58 |
| A |  |  | H | W101 | O | 3.25 |
| A |  |  | H | W101 | CB | 3.27 |
| A |  | CB | H | R99 | NH1 | 3.60 |
| A |  |  | H | W101 | CE3 | 3.35 |
| A |  |  | H | W101 | CZ3 | 3.67 |
| A | D122 | N | H | W101 | CE3 | 3.63 |
| A | Y123 | CD1 | H | W101 | CZ2 | 3.54 |
| A |  |  | H | W101 | CH2 | 3.66 |
| A |  | CE1 | H | W101 | CE2 | 3.60 |
| A |  | CE2 | H | D31 | OD2 | 3.60 |
| A |  |  | H | D31 | CB | 3.53 |
| A |  |  | H | D31 | CG | 3.21 |
| A |  |  | H | D31 | OD1 | 3.29 |
| A |  | CZ | H | D31 | CB | 3.66 |
| A |  | OH | H | D31 | OD2 | 3.35 |
| A |  |  | H | D31 | CB | 3.41 |
| A |  |  | H | D31 | CG | 3.51 |
| A | R125 | CZ | H | D31 | OD1 | 3.56 |
| A |  | NH1 | H | D31 | CG | 3.67 |
| A |  |  | H | D31 | OD1 | 2.71 |
| A |  | NH2 | H | S30 | CB | 3.57 |
| A |  |  | H | S30 | OG | 3.24 |
| A |  |  | H | Y54 | CD1 | 3.24 |
| A |  |  | H | Y54 | CE1 | 3.40 |
| A | D49 | CG | L | Y93 | OH | 3.50 |
| A |  | CD2 | L | Y93 | CZ | 3.59 |
| A |  |  | L | Y93 | OH | 2.53 |
| A | A52 | O | L | L92 | O | 3.23 |
| A |  | CB | L | Y93 | CD1 | 3.66 |
| A |  |  | L | Y93 | CE1 | 3.63 |
| A |  |  | L | L92 | O | 3.29 |
| A | I54 | CD1 | L | H94 | CG | 3.51 |
| A |  |  | L | H94 | CE1 | 3.24 |
| A |  |  | L | H94 | CE1 | 3.58 |
| A | G119 | N | L | L92 | CD1 | 3.67 |
| A |  | O | L | Y91 | CD2 | 3.35 |
| A |  |  | L | Y91 | CE2 | 3.29 |
